# The linea aspera as a guide for femoral rotation after tumor resection: is it directly posterior? A technical note

**DOI:** 10.1007/s10195-016-0399-6

**Published:** 2016-03-25

**Authors:** Ahmed Hamed Kassem Abdelaal, Norio Yamamoto, Katsuhiro Hayashi, Akihiko Takeuchi, Shinji Miwa, Ahmad Fawaz Morsy, Yoshitomo Kajino, Hiroyuki Tsuchiya

**Affiliations:** 1 Department of Orthopedic Surgery, Graduate School of Medical Science, Kanazawa University, 13-1 Takaramachi, Kanazawa, Ishikawa 921-8641 Japan; 2 Department of Orthopedic Surgery, Faculty of Medicine, Sohag University, Nasser city, 82524 Sohag, Egypt

**Keywords:** Linea aspera, Tumor resection, Intraoperative guide for posterior

## Abstract

**Background:**

The linea aspera is the rough, longitudinal crest on the posterior surface of the femoral shaft. Most orthopedic surgeons depend on the linea aspera as an intraoperative landmark identifying the true posterior aspect of the femur. We investigated the position of the linea aspera to verify whether the surgeon can rely on this accepted belief.

**Material and method:**

One hundred and thirty-three femora from 73 patients were evaluated. Four CT cuts were done of the mid femur, and we measured the angle of rotation of the linea aspera at each cut.

**Results:**

The linea aspera was externally rotated in most femora evaluated; average angles of rotation were 15.4°, 14°, 11.7°, and 11.5° at 10, 15, 20, and 25 cm from the intercondylar line, respectively. The angle of rotation of the linea aspera was positively correlated with femoral neck anteversion angle and negatively with age.

**Conclusion:**

The linea aspera is exactly posterior in a minority of individuals, while it is externally rotated to varying degrees in the majority of individuals. The degree of rotation was positively correlated with femoral neck anteversion angle, and negatively with age. To avoid implant malrotation, accurate estimation of the rotation angle should be determined preoperatively.

**Level of evidence:**

Level IV.

## Introduction

The linea aspera is the rough, longitudinal, irregular crest on the posterior surface of the shaft of the femur. It is formed by the joining of lateral and medial lips, which may be separated by up to 10 mm [1]. It is divided distally into medial and lateral supracondylar ridges. Proximally, its lateral lip continues as the gluteal tuberosity, while the medial lip is further divided into the two separate spiral and pectineal lines. The spiral line is the origin of the vastus medialis muscle and it runs medially towards the lesser trochanter. The pectineal line is the insertion for the pectineus muscle, which is located lateral and superior to it [1]. Radiographically, the linea aspera consists of two axially oriented parallel lines superimposed on the middle third of the posterior surface of the shaft of the femur [2, 3]. Most anatomical textbooks and radiological studies describe the linea aspera as a “posterior” or “midline” structure. Most orthopedic surgeons thus depend on its position as an intraoperative guide for determining the true posterior, especially when no other anatomical references are available (e.g., tumor resection of either proximal or distal parts of the femur). In this study, we investigated the rotation of the linea aspera using CT scans to determine whether orthopedic surgeons can rely on the linea aspera as a valid anatomical landmark denoting the true posterior position.

## Materials and methods

The orthopedic records of 73 patients who underwent total hip arthroplasty (THA) in our department from January 2005 to May 2014 were reviewed. Patients with a history of previous femoral fractures were excluded. There were 47 males and 26 females, with an average age of 48 ± 14.3 years (range 12–79). From these 146 femora, 13 were excluded because only postoperative CT scans of the femur were available, leaving a total of 133 femora (68 right/65 left)to be included in the study. Fifty-nine were from healthy limbs that did not undergo surgery and 74 were from limbs that were operated upon, having undergone THA.

Using EV Insite version 3.1.1.205 and AquariusNet Viewer V4.8.85 software, we built 3D models of the femora. Four CT cuts of the femoral midshaft for each femur were obtained. The reference level was considered to be at the widest intercondylar line of the femur and the posterior condylar line at that level indicated the true posterior position. We determined the location of the linea aspera at 10, 15, 20, and 25 cm from this reference level by:

1. Drawing a posterior condylar line (A) at the widest intercondylar distance and its perpendicular (B) indicating direct posterior (Fig. 1a).

**Fig. 1:**
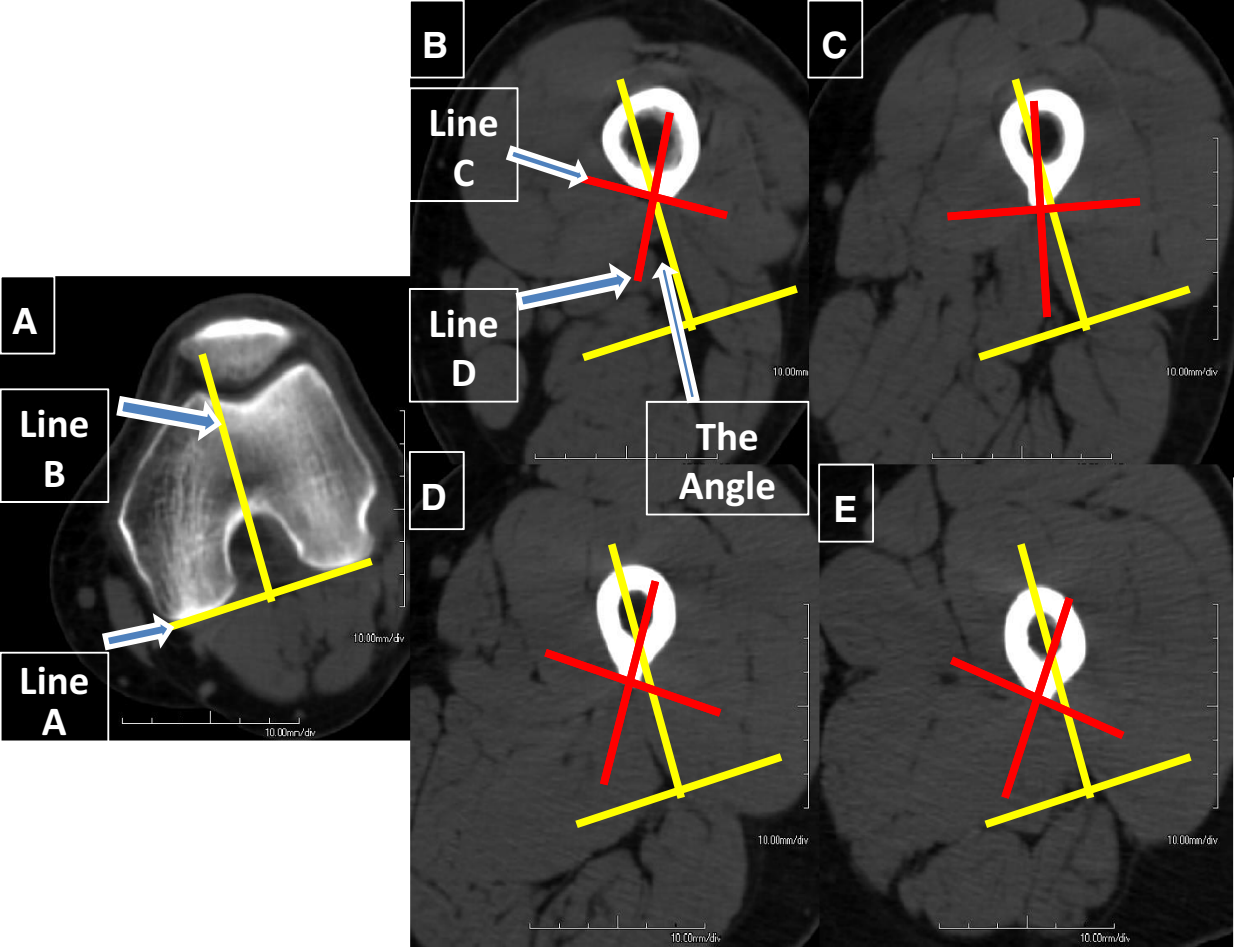
**a** Reference level (*line A*) and intersection between the posterior condylar line and its perpendicular (*line B*). **b** Tangent to lips of linea aspera (*line C*) and its perpendicular (*line D*). ARLA is the angle between *B* and *D*. **c**–**e** The ARLA measured at different levels
2. Drawing a line (C) tangential to both lips of the linea aspera.
3. Drawing a line (D) perpendicular to line (C) and measuring the angle between line (D) and line (B). This angle is the angle of rotation of linea aspera (ARLA) (Fig. 1b–e). By performing these steps at each level, an accurate estimation of the ARLA is obtained.

We only evaluated the pre-operative CT scans for the operated limbs in addition to the CT scan of the contralateral limb which had not been operated on.

## Results

The ARLAs at different levels are shown in Table 1. There was no statistically significant difference (Student *t* test) between measurements in males and females, between right and left sides, or between limbs which had undergone THA and those which had not.

**Table 1:** Minimum, average, standard deviation (*SD*), and the maximum values of age, femoral length, femoral neck anteversion angle, and linea aspera rotation angle (*ARLA*) at 10, 15, 20, and 25 cm levels

Value	Age (years)	Femoral length (cm)	Neck anteversion angle (°)	ARLA (°) at different levels			
				At 10 cm	At 15 cm	At 20 cm	At 25 cm
Minimum	12	34.5	−12	0	0	0	0
Average	48	40.4	20.4	15.4	14	11.7	11.5
SD	14.3	24.4	13.5	8.1	9.4	8.8	10.8
Maximum	79	49.9	57	30	37	35	42

The ARLA was positively correlated (moderate) with femoral neck anteversion angle, *r* = 0.1, 0.31, 0.35, and 0.29, at the 10, 15, 20, and 25 cm levels, respectively (*p* < 0.01).

A weakly negative correlation between the ARLA and age was statistically significant at the 10, 15, 20, and 25 cm levels: *r* = −0.17, −0.21, −0.106, and −0.114 (*p* < 0.05). The correlation between ARLA and femoral length was statistically insignificant (*p* > 0.05) (Table 2).

**Table 2:** Summary of correlations between ARLA and age, femoral length, and femoral neck anteversion

Level	Age *r* (*p* value)	Femoral length *r* (*p* value)	AV angle *r* (*p* value)
At 10 cm	−0.172 (0.05)	−0.049 (0.578)	0.1 (0.01)
At 15 cm	−0.213 (0.015)	0.006 (0.948)	0.314 (<0.001)
At 20 cm	−0.106 (0.007)	−0.044 (0.618)	0.353 (<0.001)
At 25 cm	−0.114 (0.03)	0 (0.799)	0.296 (0.005)

**Fig. 2:**
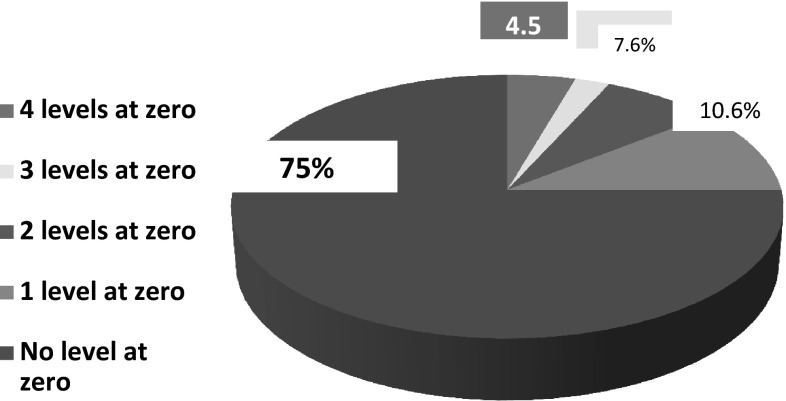
*Pie chart* represents the percentage of cases whose measurements are equal to zero (exact posterior location)

Of the 133 femora, only six showed the linea aspera being exactly posteriorly located at all four levels, representing 4.5 % of the total number of femora examined. In 75 % of the femora (99/133), the linea aspera was not shown to be exactly posterior at any of the four levels measured (Fig. 2).

## Discussion

The linea aspera has been of surgical significance for both orthopedic and vascular surgeons. The direct posterior as well as transfemoral surgical approaches are dependent on the linea aspera as a fixed anatomical landmark during the surgical procedure [4, 5]. Additionally, arthroplasty surgeons concerned with the cardiopulmonary complications of fat embolism are careful not to disrupt the venous drainage system located along the linea aspera, thereby reducing the risk of intraoperative embolism [6, 7].

The main nutrient artery to the femur passes through the linea aspera [8]; it may be in the medial or lateral lip, or even in between [9]. Yamamoto et al. [10] noted that either one or two additional large-sized nutrient vessels from the perforating branches of the profunda femoris artery also enter at different points along the linea aspera of the femur.

Radiographically, the linea aspera appears as two narrow, axially oriented, parallel lines named the “track sign” by Pitt [11] who believed it to represent the linea aspera–pilaster complex. However, other radiologists, i.e., Gheorghiu and Leinenkugel [12], claimed that the “track sign” could be readily confused with the pathological “flame sign” of Paget’s disease, leading to unnecessary investigations [13].

Many morphological studies have investigated the shape and radiographic appearance of the linea aspera. Polguj et al. [13] studied 90 human femora and suggested a four-category classification system for the shape of the linea aspera; they classified the shape of the linea aspera as straight with parallel lips, concave, inverted or variable types.

In orthopedic tumor surgery, the linea aspera is of particular importance, as after tumor resection from the proximal or distal ends of the femur, it is critical to maintain the rotational orientation of the femur during reconstruction. It is essential that malrotation be avoided during reconstruction with a tumor prosthesis as the implant has to be optimally implanted and positioned to match the femoral neck anteversion and/or knee joint orientation.

In their prestigious textbook “Musculoskeletal cancer surgery”, Malawer and Sugarbaker [14] stated that after tumor resection, “The linea aspera is the only remaining anatomical guideline for proximal and distal femur endoprosthetic replacements”; most orthopedic tumor surgeons believe this statement. This assertion was never addressed in earlier literature and no study has ever questioned whether the linea aspera is truly exactly posterior or not, despite many studies investigating the morphology of the linea aspera.

**Fig. 3:**
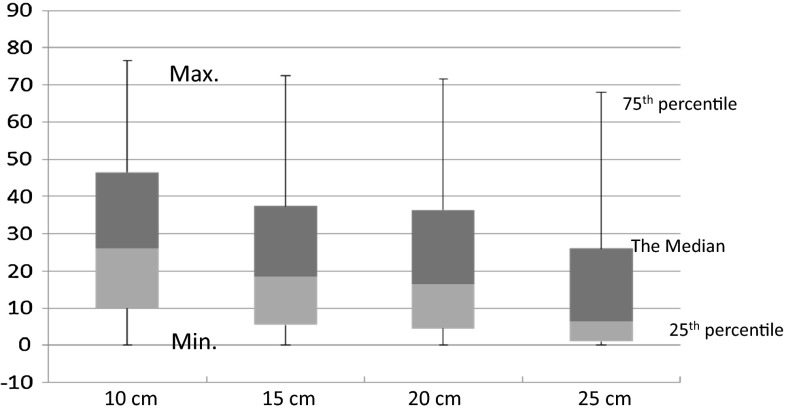
*Box plot* chart represents the distribution of values of the ARLA. The minimum, 25 percentile, median, 75 percentile and maximum values are represented for ARLA at each measured level. This chart shows the widest distribution of values at the first level (10 cm), with the values showing a gradual central tendency until the fourth level (25 cm)

The ARLA was found to be mildly externally rotated, i.e., counterclockwise on the right side and clockwise on the left side, by average angles of 15.4°, 14.0°, 11.7°, and 11.5° at 10, 15, 20, and 25 cm levels from the intercondylar reference level, respectively. Knowing that the average femoral length in our study was 40.4 cm, we can state that the 10-cm level is at the junction between the lower fourth and upper three-fourths of the femur, the 15 cm level is slightly proximal to the junction of the distal and middle thirds, the 20 cm level is at the midshaft, and the 25 cm level is approximately at the junction of the middle and proximal thirds. Values of ARLA have a wide range of variation in the distal femur with gradual decrease in the more proximal levels (Fig. 3).

Although the angle measurements may not appear large, when related to femoral neck anteversion angle, whose average was 20.4° in our cases, they are significant. If the surgeon neglects this external rotation angle and adjusts the prosthetic femoral neck anteversion based on the assumed posterior or zero position of the linea aspera, and the relationship between the ARLA and anteversion angle of the prosthesis is not taken into consideration, it is possible to implant the tumor prosthesis in a position of relative retroversion of the femoral neck (i.e., decreased anteversion).

It may be necessary for many tumor patients to eventually undergo a conversion to a THA after initial hemiarthroplasty. It is especially important in this circumstance that the femoral neck anteversion angle be accurately determined and successfully recreated at initial surgery, otherwise the acetabular cup may need to be positioned in relative retroversion to match the malaligned femoral neck. This may result in eventual dislocation of the prosthetic femoral head from the acetabular cup. Lewinnek et al. [15] described a safe zone for cup positioning as anteversion of 15 ± 10° and abduction of 40 ± 10°. McCollum and Gray [16] considered the safe range of cup placement to be 30–50° of abduction and 20–40° of flexion from the horizontal, while Dorr and Wan [17] considered cup malposition as anteversion of less than 15° or more than 30° and an abduction angle of 55° or more. Despite these guidelines for cup placement, the risk of dislocation remains a concern for the surgeon during the hemiarthroplasty procedure and even more so when converting to THA. This concern is compounded by the difficulty in radiographically assessing femoral rotation and anteversion [18].

Similarly, in distal femoral reconstruction, the surgeon cannot neglect the linea aspera external rotation angle and implant the distal femoral prosthesis assuming a zero position of the linea aspera. When the limb returns to its resting position, the femoral component may be internally rotated to a degree equal to the external rotation angle of the linea aspera.

Although these are theoretical consequences of neglecting of the ARLA, the actual outcome may be less remarkable. In total knee arthroplasty using a distal femoral prosthetic replacement, most prosthetic replacements are constrained, thus minimizing the effect of internal rotation of the femoral component. However, no-one can predict the possible long-term effect of subtle implant malrotation which may lead to loosening of the implant or other biomechanical effects on the implant. Therefore, it is important for orthopedic tumor surgeons to evaluate the orientation of the linea aspera in every case.

In our study, only six femora had an exact posterior position of the linea aspera at all four measured levels, representing 4.5 % of the total number of femora. In 74 % of the femora (99/133), the linea aspera was never in exactly posterior position at any of the four levels. The linea aspera was exactly posterior at two and three levels in only 7.6 and 2.3 % of the femora, respectively. These data do not support the conventional assertion that the linea aspera is positioned exactly posterior.

The lowermost measured level, 10 cm from the reference line, showed the largest mean of the ARLA (15.4 ± 8.1°), suggesting the importance of accurately measuring the ARLA when planning reconstruction using a distal femoral prosthesis. The mean ARLA decreases with ascending levels up the femur, where at the highest measured level it is 11.5 ± 10.8°. The orthopedic tumor surgeon must be aware of this when selecting the length of intended distal femoral prosthesis based on the level of intended resection.

The ARLA was positively correlated to femoral neck anteversion angle and was moderately correlated at the second, third and fourth measured levels of the linea aspera, which represent the mid to proximal shaft region. It was found to be weakly negatively correlated to age at the measured levels. No significant correlation was found between the ARLA and gender, femoral length, or side. We think that accurate estimation of the rotation of the linea aspera should be an important step in preoperative planning for femoral reconstruction. Evaluating its rotation in relation to the posterior condylar line will help the surgeon avoid malrotation and potential implant failure.
